# Gene Expression Control by Glucocorticoid Receptors during Innate Immune Responses

**DOI:** 10.3389/fendo.2016.00031

**Published:** 2016-04-19

**Authors:** Andre Machado Xavier, Aparecida Kataryna Olimpio Anunciato, Tatiana Rosado Rosenstock, Isaias Glezer

**Affiliations:** ^1^Department of Biochemistry, Escola Paulista de Medicina, Universidade Federal de São Paulo, São Paulo, Brazil; ^2^Department of Physiological Science, Santa Casa de São Paulo Medical School, São Paulo, Brazil

**Keywords:** acute-phase response, cortisol, gene expression, inflammatory diseases, innate immune response, GRE, SEGRAs, transrepression

## Abstract

Glucocorticoids (GCs) are potent anti-inflammatory compounds that have been extensively used in clinical practice for several decades. GC’s effects on inflammation are generally mediated through GC receptors (GRs). Signal transduction through these nuclear receptors leads to dramatic changes in gene expression programs in different cell types, typically due to GR binding to DNA or to transcription modulators. During the last decade, the view of GCs as exclusive anti-inflammatory molecules has been challenged. GR negative interference in pro-inflammatory gene expression was a landmark in terms of molecular mechanisms that suppress immune activity. In fact, GR can induce varied inhibitory molecules, including a negative regulator of *Toll*-like receptors pathway, or subject key transcription factors, such as NF-κB and AP-1, to a repressor mechanism. In contrast, the expression of some acute-phase proteins and other players of innate immunity generally requires GR signaling. Consequently, GRs must operate context-dependent inhibitory, permissive, or stimulatory effects on host defense signaling triggered by pathogens or tissue damage. This review aims to disclose how contradictory or comparable effects on inflammatory gene expression can depend on pharmacological approach (including selective GC receptor modulators; SEGRMs), cell culture, animal treatment, or transgenic strategies used as models. Although the current view of GR-signaling integrated many advances in the field, some answers to important questions remain elusive.

## Introduction

**Figure 1:**
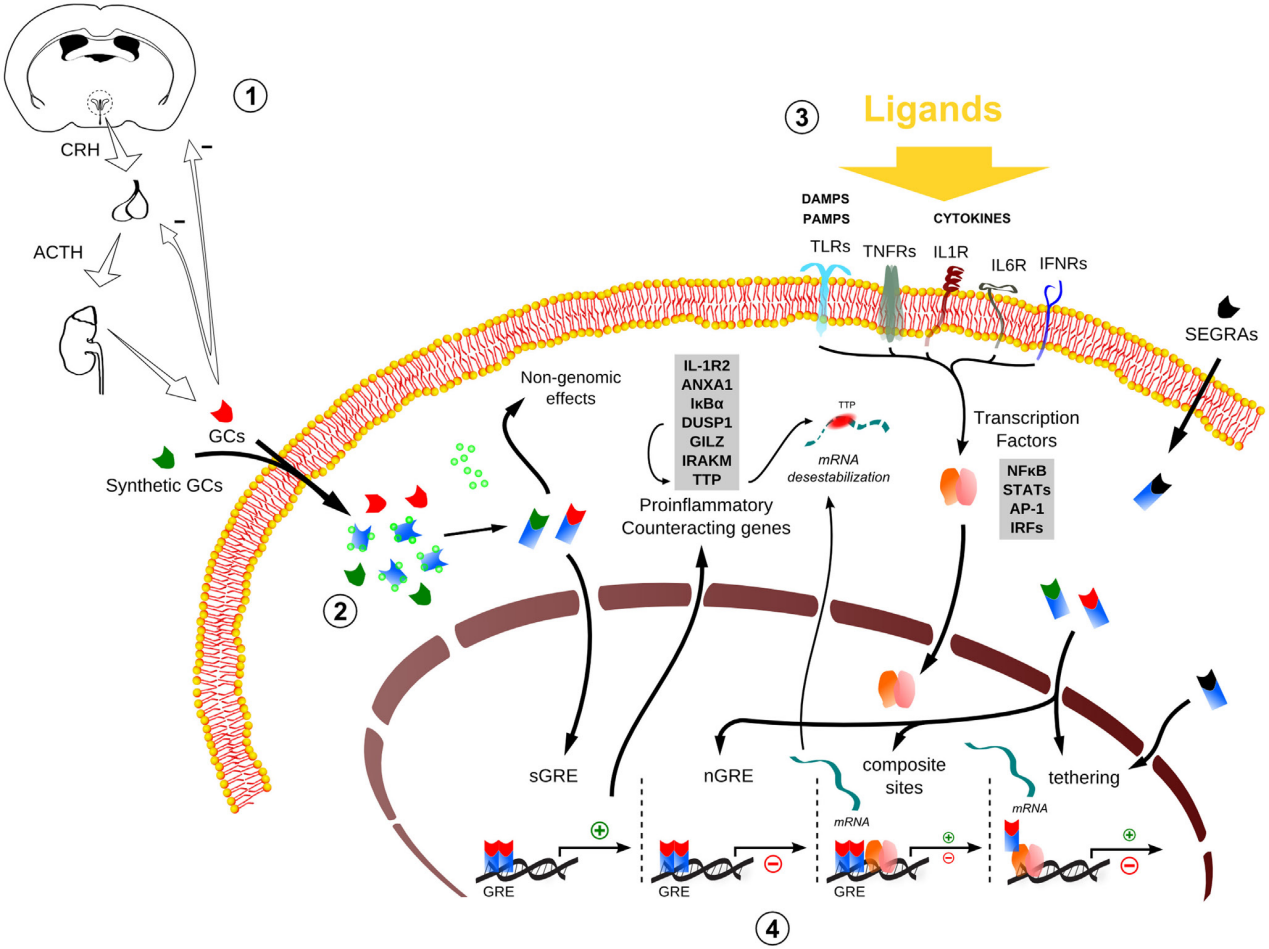
**Global scheme of glucocorticoid signaling and transcriptional mechanism during inflammation**. 1. Hypothalamus–pituitary–adrenal (HPA) signaling cascade upon stressors. CRH, corticotrophin-releasing hormone; ACTH, adrenocorticotropic hormone; GCs, glucocorticoids. 2. The endogenous/synthetic GCs bind to glucocorticoid receptor (GR) and can act in two ways: non-genomic effects in cytoplasm or translocation into the nucleus, resulting in the modulation of the transcriptional responses (for example, the transactivation of anti-inflammatory genes). Alternatively, selective glucocorticoid receptor agonists (SEGRAs) can act majorly through tethering mechanism. 3. In the context of an inflammatory scenario, cytokines, DAMPS, and PAMPs bind to their respective receptors and activate pro-inflammatory transcription factors (TFs). These TFs translocate to the nucleus and increases the activity at pro-inflammatory genes promoters by GC–GR complex (composite sites, tethering, or compete for DNA-binding sites – not shown). 4. The four main transcriptional mechanisms involved in the inflammatory response: sGRE, nGRE, binding to composite sites and tethering. In the first two modes (sGRE and nGRE), the GC–GR complex modulates the transcription in a GRE-dependent manner activating or repressing genes, if accessible. In the last two modes (composite site and tethering), the GC–GR complex is recruited to GRE sites modulating gene expression in conjunction with TFs (composite site) or interacting directly with TFs (tethering) or coactivators (not shown). Please refer to main text for more details.

An inflammatory reaction relies on both fast triggering and tight control over intensity. Failure on fine-tuning immune cells activation and pro-inflammatory signaling can lead to unnecessary expended energy and tissue damage. Endogenous glucocorticoids (GCs), as cortisol in human and corticosterone in rodents, are key hormones produced by the adrenal cortex that regulate innate immune responses. Pioneering work showed that a hormone from adrenal cortex was necessary to keep adrenolectomized animals alive after bacterial challenge (1), while a specific steroidal corticoid reversed the effects associated with adrenolectomy (2). These early studies suggest either these hormones (i.e., GCs) are necessary to mount an efficient self-defense response or counteract the aggressive side effects of this crucial reaction. While the first hypothesis will be discussed later, the second statement received attention when Phillip Hench assumed that arthritis remission could be related to high GCs blood levels. The later was verified to be true, as demonstrated by the reduction of rheumatoid arthritis symptoms upon treatment with cortisone [reviewed in Ref. (3, 4)]. An important step toward a molecular mechanism was the involvement of gene expression description in GCs anti-inflammatory effects, specifically the synthesis of an inhibitory protein or peptide (5–7). During the subsequent years, the main anti-inflammatory mechanism associated with GCs was the synthesis of Lipocortin 1 (Annexin A1; ANXA1), a phospholipase-A2 inhibitory protein that prevents the production of downstream inflammatory mediators prostaglandins and leukotrienes [reviewed in Ref. (3)]. The molecular cloning of steroid receptors increased the knowledge regarding GC receptor (GR) binding to the DNA and transcriptional control through GC response elements (GREs). These DNA sequences can mediate transactivation, as described above for ANXA1, or repression as well [reviewed in Ref. (8)]. Different modalities of GR interference in inflammatory signaling were reported, but one particular mechanism was considered more relevant to the understanding of GR functions during inflammation. This pathway drived the development of the concept of repression through tethering, which involves GR inhibitory physical interactions with nuclear activators of pro-inflammatory genes transcription (9–11). More importantly, this alternative paradigm opened the door to include other regulatory models and explore novel ligands with dissociated effects called selective GR agonists (SEGRAs) (also called dissociated GR ligands, and selective GR modulators; SEGRMs – specially in case of non-steroidal molecules) (12). GCs are known to interfere with innate immune signaling that promotes gene expression through engagement of *Toll*-like receptors (TLRs) and cytokine receptors, which leads to activation of transcription factors: nuclear factor (NF)-κB, activator protein (AP)-1, signal transducers and activators of transcription (STATs), interferon regulatory factors (IRFs), and others (13–16) (Figure 1). Here, we will discuss the perspectives of GR-mediated transcriptional activation or repression through varied mechanisms during the course of innate immune responses. We also aim to contextualize the different models of transcriptional control associated with GCs, which do not only suppresses inflammatory signaling, and point key discrepant observed outcomes and their interpretations.

## Glucocorticoid Signaling and Transcriptional Activity

The secretion of endogenous GCs results from hypothalamic–pituitary–adrenal (HPA) axis activation. Circadian, stress-related sensory information and inflammation trigger parvocellular corticotropin-releasing hormone (CRH)-secreting neurons in the paraventricular nucleus (PVN) of the hypothalamus. Once CRH reaches the anterior pituitary, responsive cells release adrenocorticotropic hormone into the bloodstream, stimulating the release of GCs from de adrenal cortex. GCs exert a negative feedback action at many levels of the HPA axis, keeping the corticoid at a physiological range (Figure 1) (17). Because steroids are water insoluble, they are transported through the blood to their target tissues mainly complexed with transcortin. GCs freely pass through cell membranes of their target cells, bind to their steroid receptors, and the complexes (steroid–receptor) translocate to nucleus, where they act as transcription factors [reviewed in Ref. (18)].

Glucocorticoid signaling depends largely on nuclear translocation and association of a hormone-bound GR dimer to 6-bp GREs (also called simple GREs), which are specific DNA sequence in the regulatory regions of target genes. The human (h) GR gene (*NR3C1*) is ubiquitously expressed and functions as a ligand-dependent transcription factor that regulates the expression of GC-responsive genes positively or negatively (18). There are two main highly homologous hGR isoforms: α and β, but others have been described as well. hGRα functions have been considerably detailed, including the complex diversity that results from alternative translation sites (19).

Glucocorticoid receptor is a modular protein, meaning that they have distinct domains: (1) the amino-terminal A/B region, also called immunogenic, functional, or N-terminal domain (NTD), and (2) the C, D, and E regions also known as structural domain, comprising the DNA-binding domain (DBD), the hinge region, and the ligand-binding domain (LBD), respectively. The NTD of the hGRα contains a major transactivation domain, named activation function (AF)-1, which plays an important role in the interaction of the receptor with molecules necessary for the initiation of transcription, such as coactivators, chromatin modulators, and basal transcription machinery. The DBD of the hGRα (region C) contains the ability to bind to GREs apart from sequences important for receptor dimerization and nuclear translocation. The LBD of the hGRα (region E) binds to GC and plays a critical role in the ligand-induced activation of hGRα. The LBD also contains a second transactivation domain, termed AF-2, which is ligand-dependent and has sequences important for receptor dimerization, nuclear translocation, and interaction with coactivators (20, 21).

Coactivators form a bridge between the DNA-bound hGRα and the transcription initiation complex and facilitate the transduction of the GC signal to RNA polymerase II activity. These include: (1) The p300 and the homologous cAMP-responsive element-binding protein (CREB)-binding protein (CBP), which also serve as macromolecular docking “platforms” for transcription factors from several signal transduction cascades, including nuclear receptors, CREB, AP-1, NF-κB, STATs, and others; (2) the p300/CBP-associated factor (p/CAF), which interacts with p300/CBP; and (3) the p160 family of coactivators (22, 23). These coactivators also have intrinsic histone acetyltransferase (HAT) activity, which promotes chromatin decondensation, and allows the transcription initiation complex of the RNA-polymerase II and its ancillary components to initiate and promote transcription (24–27).

The ligand-activated hGRα can modulate gene expression independently of binding to GREs, by interacting as a monomer with other transcription factors, such as AP-1, NF-κB, p53, and STATs (11, 28, 29). This GR nuclear action is independent of DNA-binding sites (DBSs) and involves modulation of transcriptional activity through direct protein–protein interaction (“tethering”) with inducible specific transcription factors by influencing their ability to stimulate or inhibit the transcription rates of the respective target genes (30). The negative regulation by tethering has been misleadingly conceived as the sole modality of “transrepression” during inflammation, which could be a general terminology for repression in *trans*, including those mediated by the recently characterized negative GREs (IR nGREs), a novel family of evolutionary-conserved *cis*-acting negative response elements that differs from simple GREs (31). The mechanism of repression by tethering has been the basis to revolutionize GR agonists pharmacological design (see SEGRAs/SEGRMs in Data Sheet S1 in Supplementary Material). In addition to that, GR and other transcription factors can compete for DBS, and there is also the model of composite regulation, in which GR interacts with other transcription factors at adjacent or overlapping DNA regulatory elements (32, 33). In sum, several modalities of GR interaction with DNA, coactivators/corepressors, and other transcription factors govern the complex transcriptional responses to GCs (Figure 1).

Post-translational modifications (PTMs) of GR are also relevant to GCs signaling. The hGR has several phosphorylation sites that typically occurs after binding to the ligand and may determine turnover, subcellular trafficking, target promoter specificity, cofactor interaction, strength and duration of receptor signaling, and receptor stability. Phosphorylation of the GR is a versatile mechanism for modulating and integrating multiple receptor functions (34, 35).

Other PTMs include ubiquitination, which also regulates the motility of GR inside the nucleus. After the binding of the ligand, the GR is destabilized and directed toward the proteasome pathway [reviewed in Ref. (18)]. Acetylation of the GR occurs after ligand-binding and prior to nuclear translocation. The acetylated GR is deacetylated by histone deacetylase 2 (HDAC2), and this deacetylation is necessary for the GR to be able to inhibit NF-κB activation (36). PTMs, including those not mentioned, offer an additional dimension of GR regulation that can be relevant depending on the inflammatory context.

## Synthetic Glucocorticoids, GR Antagonists, and Transgenic Animals: What Do They Inform Us about Endogenous GCs/GR Functions during Inflammation?

Two basic approaches to interrogate GR functions include mimicking the signaling with agonists, or abolishing the signaling by inhibiting endogenous GCs synthesis or blocking the receptor with antagonists. The genetic counterparts of these strategies are transgene overexpression and genetic deletion/loss-of-function mutations at the level of GR or GCs metabolism. Revealing GR roles is a challenging task, since the outcomes can vary depending on timing (early or delayed), duration (acute or chronic), GCs levels (“physiological” or “supraphysiological”), or tissue/cell type and even species. We provide Data Sheet S1 in Supplementary Material to complement information for readers not familiar with GR agonists, antagonists, and transgenic mice, and briefly point out the limitations for each strategy.

Transgenic mice have already provided valuable information about GR signaling [reviewed in Ref. (37)], including the demonstration of cell-specific GCs signaling contribution during inflammation using promoter-driven Cre-*lox* P recombination, or the evaluation of GR signaling in dimerization deficient receptor knock-in mice (GR^dim^). Dissociated agonist (SEGRM) Compound A also have proved that targeting tethered transrepression efficiently promote anti-inflammatory effects (38), corroborating GR^dim^ mice data. However, this simplified model have been questioned (39), and pharmacological investigation of GR-mediated gene transactivation showed that depending on the gene selected for analysis, a given steroid can behave as an antagonist, partial agonist, or full agonist (40). Due the complexity of GR DBSs, ligand-induced conformation changes, PTMs, and protein–protein interactions, a complete landscape of GCs transcriptional control during inflammation will require combined efforts and massive data acquisition to help define new cutting edge therapy rationale. In order to provide additional information about global gene expression in different immune cells, we provide a Table S1 in Supplementary Material comprising the main findings according to the indicated GR agonists (Table S1 in Supplementary Material).

## GR Repression on Pro-Inflammatory Genes

**Figure 2:**
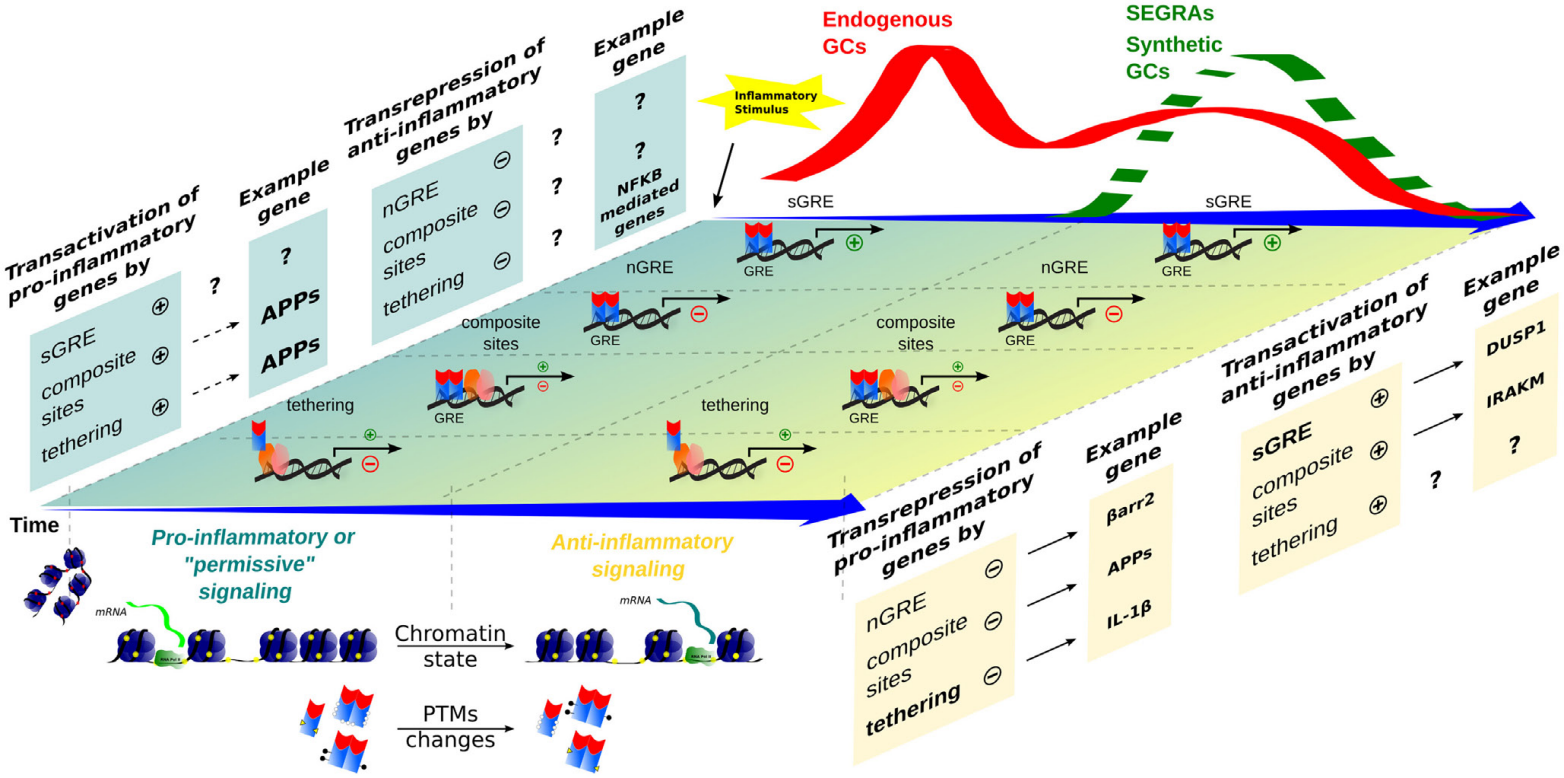
**Time-dependent evolution of inflammatory responses as orchestrated by multiple GR-dependent mechanism**. At the first moment, after an inflammatory stimulus, acute-phase proteins (APP) and other genes are transcribed through transactivation, contributing to a pro-inflammatory response that correlates with a peak of endogenous GCs levels (red wave); examples of the transcriptional modalities are still poorly described. In a second moment, a subsequent endogenous GCs wave, or administration of synthetic GCs and SEGRAs (green dashed wave), correlates with the prevalence of anti-inflammatory response governed by transactivation (anti-inflammatory genes)/transrepression (pro-inflammatory genes) mechanisms. A decreased expression of pro-inflammatory genes, for instance, IL-1β (tethering) and possible C1q (nGRE), reinforce the anti-inflammatory modality ruled by sGRE mode (DUSP1, GILZ, etc.). No positive tethering mechanism was described for anti-inflammatory genes. In contrast, IRAK-M induction through composite site with NF-κB has been reported (see main text). The chromatin remodeling and different PTMs are present in both phase of inflammatory response, offering the relevant protein interactions and DNA-binding sites for GRs/TFs.

Didactically, GR signaling may be divided into genomic (involving transcription regulation) and non-genomic. The later is faster and less characterized. Although probable, there are no strong evidences that GR engage relevant non-genomic signal transduction to repress pro-inflammatory signaling during innate immune responses (41, 42). GR genomic mechanisms that inhibit pro-inflammatory signaling include: (1) direct transcription of genes that will negatively interfere with pathways involved in the synthesis of inflammatory mediators; (2) direct repression of genes that contribute to immune cells activation; (3) negative interference in transcriptional events engaged by transcriptions factors that transduce pro-inflammatory signals; and (4) synergism between GR and other transcription factors activated during inflammation, ultimately promoting the induction of “anti-inflammatory” gene products. Examples of the first mechanism involves transcription of *ANXA1*, *NFKBIA* (IκBα), *DUSP1* (MKP-1), *GILZ*, and *ZFP36* (*TPP*). IκBα induction by GCs highjacks nuclear NF-κB, while MAP kinase phosphatase DUSP1 inactivates p38 kinase pro-inflammatory signaling, and tristetraproline (TPP) can destabilize many cytokine transcripts [reviewed in Ref. (43)]. A recent study showed that DUSP1 promotes activation of TPP destabilizing activity on *Tnf*, *Il1b*, and many other pro-inflammatory transcripts (44). For GILZ, we suggest a dedicated review (45). While direct repression (second mechanism) through nGREs during inflammation is not well characterized, some repressed genes associated with GCs anti-inflammatory effects through IR nGRE have been identified [*C1qb*, *C3*, *Il6*, etc., Ref. (31)]. However, an independent group was unable to recognize IR nGRE enrichment in their dataset (46). GR obstruction on the transactivation of pro-inflammatory genes activated by NF-κB/AP-1 (third mechanism) has been largely attributed to tethered transrepression, and to a lesser extent to composite sites or GR competition for DBS or limited coactivators (39, 43, 47). In the case of tethering, GR can associate with NF-κB and prevents the binding of IRF3 or positive transcription elongation factor b to the promoter. Conversely, GR recruits GRIP1 when tethered to AP-1, an event that may depend on nuclear thyroid hormone receptor interactor (48). We may expect that a “positive” GR tethering, recruiting coactivators, or activating the basal transcription machinery operates the expression of anti-inflammatory genes (fourth mechanism). It is not always clear if this is the case for some of the genes mentioned in the first mechanism. IRAK-M (*IRAK3*), which can block major TLRs pathways effectively, was recently described as a GR-induced gene through cooperation with NF-κB sites (49). Priming of chromatin state or presence and activity of coactivators/corepressor may impact greatly how GR modulates genes expression (50–53). We assume that all these transcriptional anti-inflammatory mechanisms prevail at late phases of an acute inflammatory reaction or when exogenous GR ligands are therapeutically delivered (Figure 2), which would be in agreement with time-dependent gene profiling assays (54).

In general, the effects of SEGRMs are less characterized; however, evidences suggest that these dissociated agonists of GR (e.g., Compound A) display an anti-inflammatory activity. In human peripheral blood mononuclear cells, Compound A inhibits the production of pro-inflammatory cytokines like TNF-α, IL-1β, and IL-6 through transrepression (55). In addition, other described SEGRMs, like ZK 216348 and Org 214007-0, showed similar anti-inflammatory effects in comparison to synthetic GC prednisolone (56, 57). According to Table S1 in Supplementary Material, there is a general agreement on a GR-mediated anti-inflammatory effect considering individual immune cells and different agonists.

Although the *in vivo* response results from a complex interplay between different cells and organs that respond differently to GCs, it is reasonable that inflammation must proceed when endogenous GCs peaks at the beginning of the reaction. It is also understandable that GR agonists are a clinically relevant option after an inflammatory burst, unless chronic treatment is considered.

## Permissive and Synergic Roles of GRs on “Pro-Inflammatory” Gene Expression

We pointed earlier the hypothesis that GCs contributes to the mounting of an efficient self-defense response. Sapolsky et al. reviewed this concept and referenced many important studies that have been historically neglected (58). When global gene expression assays became available, the fact that some genes escape GR suppression was not highlighted, since anti-inflammatory effects have always been expected. Several studies employing GR agonists or antagonists showed that various acute-phase proteins (APPs) such as serum amyloid A (SAAs), lipocalin 2 (LCN2), pentraxin 3 (PTX3), ceruloplasmin (CP), etc., are highly dependent on concomitant inflammatory stimulus and GR signaling (46, 59–62). Interestingly, induction of *Lcn2* and *Ptx3* genes by Gram-negative lipopolissacharyde (LPS) is increased by GCs and depends on IκBζ (63). Frequently, APPs pro- or anti-inflammatory nature is not clearly identified. The acute-phase response is defined as an acute inflammatory response involving non-antibody proteins whose concentration in the plasma increase in response to infection or injury of homeothermic animals (64). As part of inflammation, APPs are products of GR signaling and important players in innate immune responses (65). Fortunately, new studies have focused on the roles of some APPs, indicating that LNC2 may regulate myeloid cell polarization to pro-inflammatory (M1) phenotype (66), or contrarily, deactivate macrophages (67) and suppress cytokines production (68). PTX3 also presents ambiguous functions, since it reinforces complement function and reduces immune cells migration to sites of inflammation (69). Oncostatin M (OSM) and its receptor are potently induced by combined LPS and GR signaling *in vivo* and *in vitro*, as shown by the use of agonists and antagonists (70). The signal transduction of this neuropoietic cytokine *via* the cognate receptor can significantly synergize with pro-inflammatory cytokines (71). It is also interesting that GCs synergism with interferon signaling and STATs have been observed (59, 60), but usually the reported effect goes in the opposite direction (72). While the interpretation of synergism between GR and pro-inflammatory stimuli must be further elucidated regarding APPs, type I interferons, and non-canonical cytokines, other evidences suggests pro-inflammatory actions for GCs.

Busillo and Cidlowski proposed a molecular framework to explain how GR mediates anti-inflammatory and pro-inflammatory effects (73). The antagonistic effects were attributed to different target immune networks: pro-inflammation in innate immune response and anti-inflammatory in adaptive response. Based on several evidences that sensors NLRP3 (inflammasome component) and TLR2 are induced by GR signaling plus a pro-inflammatory trigger, the authors proposed that GCs prepares and reinforces the immune system to respond to pathogens and injury. The interpretation on NLRP3 and TLR2 inductions by GR/pro-inflammatory combination demands caution, because increased IL-1β levels and engagement of TLR signaling depends on multiple levels that can vary over the time. Their relevance must be evaluated by collectively checking if *IL1B* transcript is reduced (transcription and mRNA stability) or if toll-interacting protein (*TOLLIP*), a repressor of TLR signaling, is also induced [reported in Ref. (54)].

It has also been proposed by Busillo and Cidlowsky that the contrasting actions of GCs may rely in different signaling properties of target cells, chromatin state (availability of GR DBS), cellular binding partners, etc. Although we do agree with this model and recommend this article for the readers interested in specific cellular responses to GCs, we do not discard that pro- and anti-inflammatory resultants coexist in the same cells as a function of time (Figure 2), GCs levels (74), and interplay with other cell types. In fact, individual immune cells treated with synthetic GCs can present anti- or pro-inflammatory responses indirectly. As demonstrated by Hodrea et al., dexamethasone can promote enhanced phagocytosis by human dendritic cells through upregulation of genes related to this function, leading to subsequent increase in pro-inflammatory response (75). However, in other immune cell types, Dexamethasone exerted anti-inflammatory effects by enhancing apoptosis or by downregulating surface L-selectin in neutrophils (76, 77). Thus, GCs effects on inflammatory cells can be variable through different mechanisms. The literature regarding the effects of SEGRAs/SEGRMs in different immune cells remains quite scarce, but as demonstrated by Pazdrak et al., the responses may diverge in the same cell type depending on the agonist, as observed in eosinophils (78). We suggest that the current view on GR transcriptional modulation would benefit from models that consider additional factors not completely available in the literature yet.

## Perspectives

Important molecular achievements have been made in terms of regulatory *cis*- and *trans*-components in GCs target genes, which are now recognized as highly heterogeneous sets. Strikingly, not all gene expression data fit in predicted models (54), pointing to unrecognized regulatory determinants. To better characterize how these genes are regulated by GR, important perspectives must be incorporated, such as evidences that this nuclear receptor occupies half-sites as monomers (79), and how different agonists provoke different PTMs on GRs and the respective consequences (12). It is also mysterious, at gene expression level, the ways GRs promote pro-inflammatory or anti-inflammatory effects. What are the exact context-dependent factors that determine predominance of well-known anti-inflammatory actions or complete resistance to GCs (80)? This field of research still awaits new breakthroughs.

## Author Contributions

All authors listed have made substantial, direct, and intellectual contribution to the work and approved it for publication.

## Conflict of Interest Statement

The authors declare that the research was conducted in the absence of any commercial or financial relationships that could be construed as a potential conflict of interest.
