# Supplementary material for: Gene Expression Control by Glucocorticoid Receptors during Innate Immune Responses
Source: Front Endocrinol (Lausanne). 2016 Apr 19;7:31. doi: 10.3389/fendo.2016.00031 (PMC4835445; doi:10.3389/fendo.2016.00031)
Supplement: Supplementary file 1 [file Table_1.pdf]

Cell Type (Analysis)	Agonist	Main Findings	References
Monocytes (Microarray)	Fluticasone propionate	Down-regulation of anti-inflammatory genes CXCL11, CCL5, FCN1 (Ficolin).<br><br>Up-regulation of IL-10, IL-1RII and MRC1 genes.	(Ehrchen et al., 2016)
Macrophages (Microarray)	Dexamethasone<br><br>LPS stimulation	Down-regulation of Pro-inflammatory genes IL-1α, IL-8, IFN-α, and IFN-β.<br><br>Increased expression of scavenger receptors.	(Boettner, Ehrhart-bornstein, & Shea, 2002)
Dentritic Cells (Microarray)	Dexamethasone<br><br>LPS stimulation	Down-regulation of Pro-inflammatory genes IL-2, CCR7.	(Vizzardelli et al., 2006)
Mastocytes (Microarray)	Dexamethasone	Up-regulation of MKP-1 gene contributing to anti-inflammatory effects.	(Kassel et al., 2001)
PBMCs - Peripheral Blood Mononuclear Cells (Proteomics)	Dexamethasone<br><br>LPS stimulation	Down-regulation of Pro-inflammatory genes MICAL3, IL-1α, IL-1β, IL-6 and IL-8.<br><br>Up-regulation of M130, fibroleukin and metalloproteinase 2 genes (anti-inflammatory effects).	(Bileck, Kreutz, Muqaku, Slany, & Gerner, 2014)
Neutrophils (Proteomics)	Dexamethasone	Down-regulation of annexin 1 (lipocortin 1), phosphoglycerate mutase and cathelicidin 1 genes.<br><br>Up-regulation of Lactotransferrin (lactoferrin) contributing to Pro-inflammatory effects.	(Beveridge, Mitchell, Brewer, Clark, & Caswell, 2008)

Epithelial Cells (Microarray)	Dexamethasone	Up-regulation of MKP-1 gene contributing to anti-inflammatory effects.	(Wu et al., 2004)
THP1- Monocytic Cell Line (Microarray)	Org 214007-0	Down-regulation of IL-6, IL-8 and MCP-1 genes.<br><br>Up- regulation of FK506, GILZ and DUSP1 genes.	(Lierop et al., 2012)
Neutrophils (Microarray)	Dexamethasone	Down-regulation of Caspase-8,TF-1 and TFAR-19 genes, contributing to anti-apoptothic effects.<br><br>Up-regulation of PPAR-γ, BPI and LC3 genes.	(Weber et al., 2006)

Supplementary Table 1 – Summary of published Microarray/Proteomics data from studies employing different immune cells and the indicated GR agonists.

References

Beveridge, J. D., Mitchell, G. B., Brewer, D., Clark, M. E., & Caswell, J. L. (2008). Altered protein expression in neutrophils of calves treated with dexamethasone. *Canadian Journal of Veterinary Research*, *72*(519), 249–252.

Bileck, A., Kreutz, D., Muqaku, B., Slany, A., & Gerner, C. (2014). Comprehensive Assessment of Proteins Regulated by Dexamethasone Reveals Novel E ff ects in Primary Human Peripheral Blood Mononuclear Cells. *Journal of Proteome Research*, *2*(i), 5989–6000.

Boettner, A., Ehrhart-bornstein, M., & Shea, J. J. O. (2002). Gene profiling reveals unknown enhancing and suppressive actions of glucocorticoids on immune cells. *FASEB Journal*, *1*(16), 61–71.

Ehrchen, J., Steinmu, L., Barczyk, K., Tenbrock, K., Nacken, W., Eisenacher, M., … Roth, J. (2016). Glucocorticoids induce differentiation of a specifically activated, anti-inflammatory subtype of human monocytes. *Blood Journal*, *109*(3), 1265–1275. http://doi.org/10.1182/blood-2006-02-001115.The

Kassel, O., Sancono, A., Kra, È., Kreft, B., Stassen, M., & Cato, A. C. B. (2001). Glucocorticoids inhibit MAP kinase via increased expression and decreased degradation of MKP-1. *The EMBO Journal*, *20*(24), 7108–7116.

Lierop, C. Van, Alkema, W., Laskewitz, A. J., Dijkema, R., Der, H. M. Van, Smit, M. J., … Dokter, W. H. A. (2012). Org 214007-0 : A Novel Non-Steroidal Selective Glucocorticoid Receptor Modulator with Full Anti- Inflammatory Properties and Improved Therapeutic Index. *Plos One*, *7*(11). http://doi.org/10.1371/journal.pone.0048385

Vizzardelli, C., Pavelka, N., Luchini, A., Zanoni, I., Bendickson, L., Pelizzola, M., … Nilsen-hamilton, M. (2006). Effects of dexamethazone on LPS-induced activation and migration of mouse dendritic cells revealed by a genome-wide transcriptional analysis. *European Journal of Immunology*, *36*, 1504–1515. http://doi.org/10.1002/eji.200535488

Weber, P. S. D., Madsen-Bouterse, S. A., Rosa, G. J. M., Sipkovsky, S., Ren, X., Almeida, P. E., … Burton, J. L. (2006). Analysis of the bovine neutrophil transcriptome during glucocorticoid treatment. *Physiological Genomics*, *28*(1), 97–112. http://doi.org/10.1152/physiolgenomics.00094.2006

Wu, W., Chaudhuri, S., Brickley, D. R., Pang, D., Karrison, T., & Conzen, S. D. (2004). Microarray Analysis Reveals Glucocorticoid-Regulated Survival Genes That Are Associated With Inhibition of Apoptosis in Breast Epithelial Cells. *Cancer Research*, *64*, 1757–1764.
