# Supplementary material for: Gene Expression Control by Glucocorticoid Receptors during Innate Immune Responses
Source: Front Endocrinol (Lausanne). 2016 Apr 19;7:31. doi: 10.3389/fendo.2016.00031 (PMC4835445; doi:10.3389/fendo.2016.00031)
Supplement: Supplementary file 2 [file Data_Sheet_1.pdf]

## Supplementary Box 1

## Synthetic glucocorticoids, GR antagonists and transgenic animals: what do they tell us about endogenous GCs/GR functions during inflammation?

*Agonists*

Agonist strength, directed biotransformation and selectivity for GRs over other steroid receptors have been the key elements to mitigate substantial side effects associated with GCs-based therapy. In consequence, the earlier generations of GR agonists, including those used in therapeutics (dexamethasone, prednisone, budesonide, etc.), addressed the basic aspects of pharmacological targeting, but failed to avoid the detrimental effects associated with GR overstimulation. For instance, Dexamethasone (DEX) shows substantial GR selectivity, and unlike endogenous GCs, the synthetic corticoid is not metabolized by the enzyme 11-β-hydroxysteroid dehydrogenase. DEX long-term treatment is known to promote serious health hazards, namely diabetes, glaucoma, HPA axis suppression, gastrointestinal ulcers, myopathies and osteoporosis (Fitch and van de Beek, 2008; Schacke et al., 2002). The possibility of dissociatie monomeric GR-mediated transrepression from dimeric GR binding to GRE sites fostered a new drug design rationale aimed specifically to restrain pro-inflammatory signaling. Since monomeric GR tethering is considered the prevalent mechanism to curb exacerbated inflammation, this new strategy can offer anti-inflammatory action without inducing or interfering with transcriptional events regulated by dimeric GR/GRE sites. The efficacy of non-steroidal SEGRMs has been broadly evaluated and reviewed, especially data regarding Compound A. This particular molecule successfully reduces inflammatory parameters and mediators *in vitro* and *in vivo* without rising glycaemia or inducing positive GRE-regulated genes (*DUSP1/MKP1*, *GILZ* and *FKBP51*). It has been hypothesized that the different mode of ligand binding to GR promotes the alternative conformations that operates the monomeric GR mode of action  (De Bosscher et al., 2010; Lesovaya et al., 2015; Sundahl et al., 2015). In

contrast, DEX and prednisolone regulate quite the same genes in acute lymphoblastic leukemia cells (Bindreither et al., 2014), suggesting that only potency is the relevant pharmacodynamical difference between the two synthetic GCs, ate least in these cells. Although agonists provides valuable information, one should keep in mind that sometimes they fail to offer realistic information regarding endogenous GCs functions.

*Antagonists and Inhibitors*

The most well-characterized anti-GC drug RU486 (mefipristone) is a GR and Progesterone receptor (PR; NR3C3) antagonist. It can be successfully used to treat excessive GC signaling in Cushing's Disease due to the high affinity of the drug to GR and lack of transactivation activity (Johanssen and Allolio, 2007; Spitz and Bardin, 1993). Partial agonist activity is a feature of this molecule (Schulz et al., 2002), but this do not preclude the use of RU486 to uncover endogenous GCs roles, especially when progesterone signaling is not a relevant interference. A more specific compound designated ORG 34517 have been shown to avoid specifically GR translocation (Peeters et al., 2008), offering a new pharmacological option. Other approaches include the inhibition of GC synthesis at the level of 11β-hydroxilation with metyrapone. These resources provide the possibility to attenuate GR signaling at varied degrees through dosing, but care must been taken with rebound HPA axis activity and overall physiological changes.

*Transgenic mice*

It is beyond the scope of this review to catalog all mutants and transgenic mice concerning GR, the reader should refer to dedicated material elsewhere (Harris, 2015). The first attempt to knock-down GR with antisense RNA expression under the control of neurofilament promoter lead to over-activated HPA axis, which excluded its use to clarify the role of GR and pro-inflammatory signaling. In oppose to that, GR-null mice dies after birth. In consequence, these mice could only be used as donors to heterologue transplantation, or as source of primary cell culture. Site-directed mutagenesis in GR lead to the identification of a modification that preserved repression, but inhibit transactivation by the receptor (Heck et al., 1994). GR dim

mice were generated by homologous recombination that mimicked the lack of GR dimerization, and can be used as a tool to interrogate transactivation dissociated from repression. The use of *Cre-lox* recombination technology provided the arsenal to interrogate GR functions in cell/tissue specific manner, and in consequence a myriad of experimental data have been generated lately regarding both *in vivo* and *in vitro* models (see Harris, 2015). Researchers must be aware that although these constructions supported viable mice, constitutive *Cre* expression and loxP-targeted excision can lead to lifetime or age-dependent adaptive changes that may modulate the overall physiology of the target organ and confound the results from acute challenges.

## References


Bindreither, D., Ecker, S., Gschirr, B., Kofler, A., Kofler, R., and Rainer, J. (2014). The synthetic glucocorticoids prednisolone and dexamethasone regulate the same genes in acute lymphoblastic leukemia cells. *BMC Genomis* 15, 662–670. doi:10.1186/1471-2164-15-662.

De Bosscher, K., Haegeman, G., and Elewaut, D. (2010). Targeting inflammation using selective glucocorticoid receptor modulators. *Curr. Opin. Pharmacol.* 10, 497–504. doi:10.1016/j.coph.2010.04.007.

Fitch, M. T., and van de Beek, D. (2008). Drug Insight: steroids in CNS infectious diseases--new indications for an old therapy. *Nat. Clin. Pract. Neurol.* 4, 97–104. doi:10.1038/ncpneuro0713.

Harris, C. (2015). "Animal Models of Altered Glucocorticoid Signaling," in *Glucocorticoid Signaling SE - 15* Advances in Experimental Medicine and Biology., eds. J.-C. Wang and C. Harris (Springer New York), 337–352. doi:10.1007/978-1-4939-2895-8_15.

Heck, S., Kullmann, M., Gast, A., Ponta, H., Rahmsdorf, H. J., Herrlich, P., et al. (1994). A distinct modulating domain in glucocorticoid receptor monomers in the repression of activity of the transcription factor AP-1. *EMBO J.* 13, 4087–95. Available at: http://www.pubmedcentral.nih.gov/articlerender.fcgi?artid=395330&tool=pmcentrez&rendertype=abstract.

Johanssen, S., and Allolio, B. (2007). Mifepristone (RU 486) in Cushing's syndrome. *Eur. J. Endocrinol.* 157, 561–569. doi:10.1530/EJE-07-0458.

Lesovaya, E., Yemelyanov, A., Swart, A. C., Swart, P., Haegeman, G., and Budunova, I. (2015). Discovery of Compound A – a selective activator of the glucocorticoid receptor with anti-inflammatory and anti-cancer activity. *Oncotarget; Vol 6, No 31*. Available at: http://www.impactjournals.com/oncotarget/index.php?journal=oncotarget&amp.

Peeters, B. W. M. M., Ruigt, G. S. F., Craighead, M., and Kitchener, P. (2008). Differential effects of the new glucocorticoid receptor antagonist ORG 34517 and RU486

(mifepristone) on glucocorticoid receptor nuclear translocation in the AtT20 cell line. *Ann. N. Y. Acad. Sci.* 1148, 536–41. doi:10.1196/annals.1410.072.

Schacke, H., Docke, W. D., and Asadullah, K. (2002). Mechanisms involved in the side effects of glucocorticoids. *Pharmacol Ther* 96, 23–43. doi:S0163725802002978 [pii].

Schulz, M., Eggert, M., Baniahmad, A., Dostert, A., Heinzel, T., and Renkawitz, R. (2002). RU486-induced glucocorticoid receptor agonism is controlled by the receptor N terminus and by corepressor binding. *J Biol Chem* 277, 26238–26243. doi:10.1074/jbc.M203268200.

Spitz, I. M., and Bardin, C. W. (1993). Mifepristone (RU 486)--a modulator of progestin and glucocorticoid action. *N Engl J Med* 329, 404–412. Available at: http://www.ncbi.nlm.nih.gov/entrez/query.fcgi?cmd=Retrieve&db=PubMed&dopt=Citation&list_uids=8326975.

Sundahl, N., Bridelance, J., Libert, C., De Bosscher, K., and Beck, I. M. (2015). Selective glucocorticoid receptor modulation: New directions with non-steroidal scaffolds. *Pharmacol. Ther.* doi:10.1016/j.pharmthera.2015.05.001.
